# Microbial growth and importance of flushing inside closed-type infusion devices during administration of lipid emulsion *in vitro* setting

**DOI:** 10.7150/ijms.60200

**Published:** 2021-06-26

**Authors:** Sachiko Omotani, Yasutoshi Hatsuda, Yasuhiro Katsui, Ayumi Asao, Hiroyuki Toujou, Keigo Ihara, Katsuji Tani, Michiaki Myotoku

**Affiliations:** Faculty of Pharmacy, Osaka Ohtani University.

**Keywords:** Total Parenteral Nutrition (TPN), lipid emulsion, closed-type infusion device, flushing, catheter-related blood stream infections (CRBSI)

## Abstract

**Background:** We investigated the extent of growth of microorganisms with simultaneous administration of lipid emulsions with infusions for Total Parenteral Nutrition (TPN), assuming that the lipid emulsions contaminated with microorganisms are stagnant in a closed-type infusion device. We also investigated if bacterial growth can be prevented in the infusion device by flushing the inside of the infusion device with saline solution after the administration of lipid emulsion from the side tube *in vitro* setting.

**Methods:** We made a preparation by adding *Escherichia coli* to the lipid emulsion and started the infusion simultaneously with the infusion solution for TPN and lipid emulsion with the piggyback method. Immediately after the completion of lipid emulsion infusion, we conducted flushing with saline solution. The volume of saline solution was none, 5, 10, or 20 mL at a flow rate of 1 mL/s. Infusion solution that was stagnant in the infusion device was collected immediately before completing the lipid emulsion infusion and 20 h after flushing, i.e., 24 h after starting the infusion for TPN, and the number of viable bacteria was determined.

**Results:** The number of viable *E. coli* increased in the infusion device of all three species used in this experiment 24 h after starting the lipid emulsion infusion without flushing. We found that bacterial growth could be prevented through flushing with saline solution after the completion of lipid emulsion infusion and flushing out the stagnant infusion solution in the closed-type infusion device.

**Conclusions:** We found that if *E. coli* was present in the closed-type infusion device, it would multiply. We also found that the number of viable bacteria varied according to the variety and internal structure of the closed-type infusion device as well as the liquid volume used for flushing, although flushing can prevent the growth of microorganisms. Proper management and manipulation of infusion is required to prevent infection.

## Introduction

The administration of lipid emulsions is not only helpful in the prevention of essential fatty acid deficiency and the supply of energy but also in the prevention of fatty liver and Total Parenteral Nutrition (TPN)-related liver damage during intravenous nutrition 1. The third edition of the Guidelines for Parenteral and Enteral Nutrition 1 states that lipid emulsions can be administered through the side tube of a central venous line during TPN implementation. However, there is a concern that lipid emulsions can be a source of infection as they are reported to be rich in nutrients, and could serve as a possible source of nutrition for microorganisms 2. According to the guidelines, it is suggested that lipid emulsions should be administered at a rate of no more than 0.1 g/kg/h 1. Moreover, according to the instructions in the 2011 Centers for Disease Control and Prevention Guidelines for the prevention of intravascular catheter-related infections 3, infusion routes for TPN without the administration of lipid emulsion should be changed at least every 7 days at intervals of 96 h or more, whereas the routes of lipid emulsion should be exchanged within 24 h. However, when the lipid emulsion administered from the side tube stagnates in the infusion device, it is not removed until the route of the main tube is changed. While some of the stagnant lipid emulsion is often flushed out with saline solution from a side tube at the end of its administration via the infusion device, the method of administration and the amount vary according to the local standards of care. It is clear from various reports that TPN, catheter insertion, or improper handling of devices are risk factors for catheter-related blood stream infections (CRBSI) infections 3,4,5.

Therefore, we investigated the extent of growth of microorganisms inside the closed-type infusion device with the simultaneous administration of lipid emulsion through the side tube of a central venous line during administration of TPN infusion considering that microorganisms contaminated the closed-type infusion device through the side tube. We also investigated whether the growth of bacteria inside the closed-type infusion device could be prevented by flushing saline solution from the side tube after the completion of lipid emulsion administration.

## Materials and methods

### Microorganisms employed

The bacterial strain used in the study was the *Escherichia coli* (*E. coli*) W 3110, that one of the causative pathogens CRBSI. Luria-Bertani (LB) medium and Pearlcore^®^ normal agar medium 'Eiken' (Eiken Chemical Co., Ltd., Tokyo, Japan) were used as the medium.

### Test solutions and devices

ELNEOPA^®^-NF No. 2 Injection (2000 mL, Otsuka Pharmaceutical Factory, Inc., Tokushima, Japan) was used for TPN infusion solution. Intralipos^®^ Injection 20% (100 mL, Otsuka Pharmaceutical Factory, Inc., Tokushima, Japan) was used for lipid emulsion, and Otsuka Normal Saline was used as the saline solution for flushing from the side tube (20 mL, Otsuka Pharmaceutical Factory, Inc., Tokushima, Japan). For flushing, 10 mL and 20 mL Luer-Slip-type syringes (NIPRO Corporation, Osaka, Japan) were used. The Terufusion^®^ Infusion Pump TE-131 (Terumo Corporation, Tokyo, Japan) was used as the infusion pump, and Terufusion^®^ Infusion Set TI-J350P (Terumo Corporation, Tokyo, Japan) was used as the infusion set. All three types of devices used were closed types system: (A) Sure Plug AD 3-way stopcock SA-TR13 (Terumo Corporation, Tokyo, Japan), (B) Top 3-way stopcock SL(360°)-F-CL (Top Corporation, Tokyo, Japan), and (C) BD connecta™ Ref394501 (Nippon Becton Dickinson Company, Ltd., Tokyo, Japan).

### Culture methods and sampling

*E. coli* was added to 5 mL of Luria-Bertani (LB) medium in sterile centrifuge tubes, and incubated at 37 °C overnight. Then, microbial cells were collected and washed with sterile phosphate-buffered saline (PBS) by centrifugation. The bacterial solution was added to the lipid emulsion so that about 10^1^-10^2^ CFU of *E. coli* were mixed in the closed-type infusion device just before the end of the lipid emulsion administration.

Using the Terufusion^®^ Infusion Pump, the drips of TPN infusion solution and lipid emulsion were started simultaneously by the piggyback method at flow rates of 2,000 mL/24 h and 100 mL/4 h, respectively. Doses were set according to the guidelines; the administration of 100 mL of 20% lipid emulsion to patients weighing 50 kg should be done over 4 h. The saline solution was used for flushing after the completion of the lipid emulsion drip (4 h after starting the TPN infusion and lipid emulsion drip). The volume of flushing was set to none, 5, 10, or 20 mL. Under aseptic conditions, 5 or 10 mL of saline solution was measured in a 10 mL syringe, and 20 mL of saline solution was measured in a 20 mL syringe, and either saline solution was manually administered at a flow rate of 1 mL/s through the side tube of a closed-type infusion devices.

The infusion solution that stagnated in the infusion device was collected: (1) immediately before completing the lipid emulsion drip and, (2) 24 h after starting the TPN infusion drip (20 h after the completion of the lipid emulsion drip). After collecting the infusion solution that stagnated inside the infusion device 24 h after starting the TPN infusion drip, the bacteria remaining inside the infusion device were collected after flushing with 20 mL of PBS. The point of time at 90 mL drip after starting the lipid emulsion drip was defined as the time (1) immediately before completing the lipid emulsion drip. All the experiments were repeated five times. Moreover, the collected samples were left to stand at 37 °C using a medium and the number of viable bacteria was measured by the colony coefficient method after 24 h.

### Enumeration of viable cells

A statistical analysis was performed using IBM SPSS Statistics Desktop Ver.21 (IBM Community Japan, Tokyo, Japan). Tukey's multiple comparison test were used to analyze the data. *P*-values of 0.05 were considered significant.

## Results

**Figure 3:**
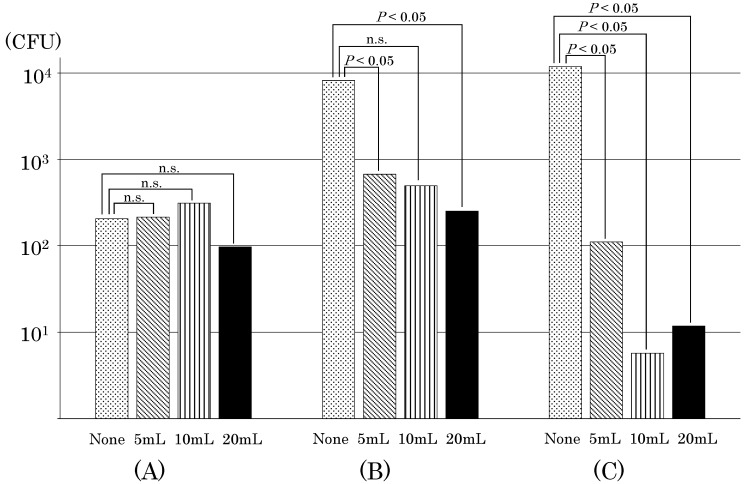
The average value of bacterial growth by flush volume for each device is shown. The total number of bacteria grown inside the device was shown, assuming that the bacteria count was 100 CFU before the end of the lipid emulsion drip. Devices were used as follows: (A) Sure Plug AD 3-way stopcock SA-TR13 (Terumo Corporation, Tokyo, Japan), (B) Top 3-way stopcock SL(360°)-F-CL (Top Corporation, Tokyo, Japan), and (C) BD connecta™ Ref394501 (Nippon Becton Dickinson Company, Ltd., Tokyo, Japan). Tukey's multiple comparison test were used to analyze the data (*P<0.05*). “n.s.” indicates no significant difference.

Table 1 shows the results of viable bacteria count inside the infusion devices over time. Table 2 shows the number of samples with zero viable bacteria inside the infusion device after 24 h (the sum of the viable bacteria count inside the infusion device 24 h after the start of TPN infusion and the residual viable bacteria count inside the infusion device collected by flushing the inside of the infusion device with PBS). Figure 3 shows the average value of bacterial growth by flash volume for each device.

Bacterial viability was confirmed in all three types of closed-type infusion devices after completing the TPN infusion without flushing. In the Sure Plug AD 3-way stopcock SA-TR13 (hereinafter referred to as the infusion device (A)), compared with that immediately before completing the lipid emulsion, the total number of bacteria inside the infusion device increased in three out of five experiments after the completion of TPN infusion. The number of viable *E. coli* in the infusion device was 77 CFU immediately before the completion of the lipid emulsion drip, but 325 CFU of *E. coli* stagnated in the infusion device 24 h after the start of TPN infusion. In addition, 61 CFU of *E. coli* were viable in the infusion device after washing with PBS, revealing that a total of 386 CFU of *E. coli* were viable in the infusion device after the completion of TPN infusion. Top three-way stopcock SL (360°)-F-CL (hereinafter referred to as the infusion device (B)) had an increasing number of bacteria in all of the five experiments. The number of viable *E. coli* inside the infusion device was 13 CFU immediately before the completion of the lipid emulsion drip, but the total number of bacteria inside the infusion device increased to 3,313 CFU after the completion of the TPN infusion drip. BD connecta™ Ref394501 (hereinafter referred to as the infusion device (C)) was also confirmed to have a significant increase in all experiments. The highest level of *E. coli* was 32 CFU immediately before the completion of the lipid emulsion drip. In addition, the number of viable *E. coli* was 14,607 CFU inside the infusion device by flushing with PBS, and *E. coli* increased inside the infusion device. On the other hand, an increase in *E. coli* was not observed in all infusion devices by flushing with saline solution after the completion of the lipid emulsion drip when compared with no flushing. In the infusion device (A), the number of *E. coli* increased in three out of five experiments when flushing with 5 mL, but the growth rate of *E. coli* was also reduced when the volume of flushing increased from 10 mL to 20 mL, and not determined three times for the total number of viable bacteria inside the infusion device by flushing with 10 mL after the completion of the lipid emulsion drip. Conversely, while in the infusion device (B) the number of *E. coli* increased in all experiments without flushing, the growth rate of *E. coli* was reduced independently depending upon the volume of flushing. By flushing with increasing volumes of 5 mL, 10 mL, and 20 mL after the lipid emulsion drip, the frequency at which viable bacteria could not be determined was rising, that is, twice, three times, respectively. The number of *E. coli* increased in all experiments without flushing in the infusion device (C). However, the growth rate of *E. coli* was reduced more than that in the infusion device (B) even by flushing with a volume of 5 mL, and an increase of *E. coli* was not observed inside the infusion device in all experiments by flushing with 10 mL or 20 mL after the completion of the lipid emulsion drip.

## Discussion

There are some reports about improper handling of catheters as one of the causes of CRBSI 6,7,8. Conversely, a closed-type infusion device, which is easy to operate for the simultaneous administration of multiple drugs, is used broadly in clinical practice. Various types of infusion devices are commercialized by a variety of manufacturers, and closed-type infusion devices are available for infection prevention. There are various reports about the requirement to disinfect the side tube of infusion device 9,10,11; however, there are still few reports on bacterial contamination in the closed-type infusion device, the requirement of flushing from the side tube after the administration of drugs, and the volume for flushing 12. The most commonly reported dose of flush overseas was 5 mL 8. On the other hand, in Japan, the dosage of flushes varies among medical institutions, and the Guidelines for Parenteral and Enteral Nutrition 1 do not provide any explanation or rationale for whether or not flushes should be performed in the line after lipid emulsion administration, which is an issue. Therefore, at present, whether to flush or not, or the dose of flush administered, tends to be influenced by the experience of nurses, as the report 7.

Therefore, we examined whether bacterial growth prevention in the closed-type infusion device could be achieved by washing out, that is, flushing, inside the closed-type infusion device through the side tube with saline solution after the administration of lipid emulsion, if bacteria contaminate the closed-type infusion device during TPN administration *in vitro* setting. In this experiment, since there is a limit to using all the causative bacteria of CRBSI, *E. coli*, which is one of the causative bacteria of CRBSI, was used. Although the incidence of methicillin-resistant *Staphylococcus aureus* CRBSI has decreased in recent years, perhaps as a result of prevention efforts, for gram negative rods, antimicrobial resistance to third generation cephalosporins among *E. coli* has increased 3.

In the present study, we compared the number of viable bacterial immediately before the completion of the lipid emulsion drip with the number of viable bacteria 24 h after starting the TPN infusion drip to examine whether bacteria grow inside the closed-type infusion device. It was elucidated that bacteria grew inside the closed-type infusion device unless flushing was performed. We also found that because flushing reduced the number of bacteria that stagnated inside the closed-type infusion device, it is possible to prevent the growth of bacteria by flushing.

In the infusion device (A), *E. coli* was still present even after flushing with 20 mL of saline solution; the highest volume of saline used in this experiment, although the number of residual viable bacteria reduced as the flush volume of saline solution increased. Moreover, in the infusion device (C), there were some cases where *E. coli* was often not observed to exist after flushing with 10 mL or 20 mL saline solution when compared with the infusion device (A); however, there were other cases where *E. coli* existed even after flushing with 20 mL saline solution. These results elucidated that we could not completely remove the infusion solution and bacteria stagnating inside the infusion device even after flushing the inside of the infusion device with saline solution. The differences in the number of viable bacteria remaining in the closed-type infusion devices are thought to depend on their structure. Regarding the side tubular part of the closed-system infusion device, because the inner structure has a complicated construction to close down, it is considered that we could not push out the infusion fluid and microorganisms stagnating in the structure part just by flushing with saline solution. When using the infusion device (B), compared with the infusion device (A) and the infusion device (C), because more viable *E. coli* existed after the completion of TPN infusion drip and inside the infusion device, it is considered that more complex the structure among the same closed-type infusion devices, the more likelihood of stagnation of the infusion solution and microorganisms.

Therefore, in the case of simultaneous administration of TPN infusion solution and lipid emulsion, and if the lipid emulsion and bacteria remain inside the closed-type infusion device even after flushing, there is a risk of an increase in the bacteria before the completion of administration of TPN infusion solution. To prevent such bacterial growth, it is necessary to flush a sufficient volume of saline solution so as remove all the lipid emulsion and bacteria from inside the closed-type infusion device. It is also important to prevent bacteria from invading the closed-type infusion device. It is necessary to aseptically prepare the infusion solution and to disinfect the parts of the connection with ethanol.

The present experiment demonstrated that it is possible to prevent nutrient infusion solution from stagnating, which may cause bacterial growth, by flushing the closed-type infusion device with saline solution or other solutions after the administration of drugs through the side tube of the closed-type infusion device. However, it was also revealed that there were differences in the number of viable bacteria after the TPN infusion drip for 24 h depending on the type of closed-type infusion device and the volume used for flushing.

In this experiment, we did not study the effect of the method of flush administration because we studied the effect of whether or not to perform the flush and the dose of flush administered after lipid emulsion administration. As a limitation of this experiment, it was also clear that the flush was performed manually and thus was greatly influenced by the individual's technique, which is similar to the report that the flushing technique is influenced by the nurse's experience and workload 7.

In the future, we consider it possible to remove nutrient solution and bacteria that have stagnated inside the closed-type infusion device and prevent infection by improving the flushing method, such as using a pulsatile flush in reported 13,14, to enhance the effect of physically cleaning the interior of the closed-type infusion device.

## Figures and Tables

**Figure 1:**
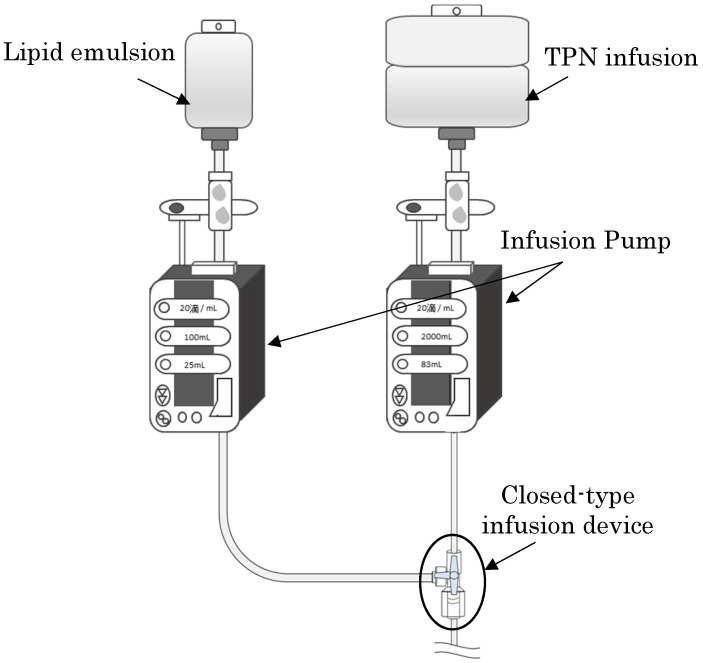
A schematic diagram of the experimental method is shown.

**Figure 2:**
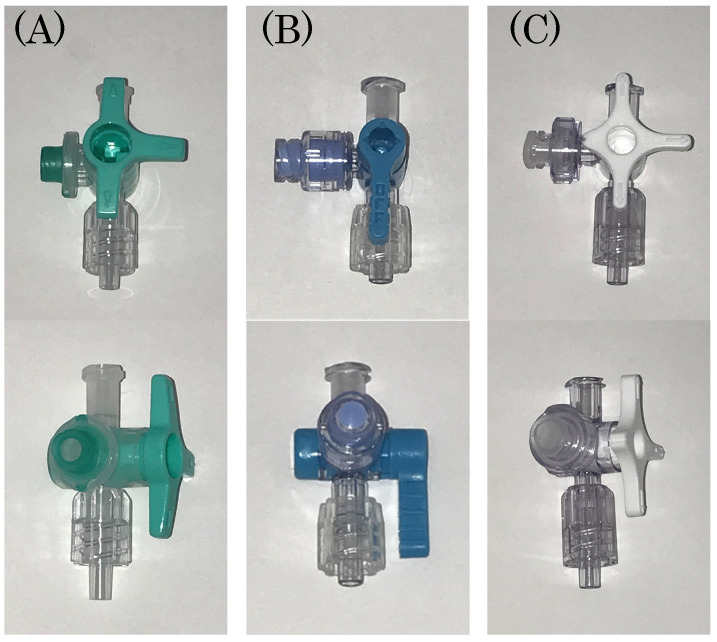
The figure of the three kinds of closed-type infusion device used is shown: (A) Sure Plug AD 3-way stopcock SA-TR13 (Terumo Corporation, Tokyo, Japan), (B) Top 3-way stopcock SL(360°)-F-CL (Top Corporation, Tokyo, Japan), and (C) BD connecta™ Ref394501 (Nippon Becton Dickinson Company, Ltd., Tokyo, Japan).

**Table 1:** Changes over time in the number of viable bacteria inside an infusion device

The volume of saline flush	Number of experiments	Type of infusion device					
		(A)		(B)		(C)	
		(1) Immediately before the end of lipid emulsion drip	(2) Total number of bacteria inside the infusion device (after completion of TPN infusion + the number of residual viable bacteria inside the infusion device)	(1) Immediately before the end of lipid emulsion drip	(2) Total number of bacteria inside the infusion device (after completion of TPN infusion + the number of residual viable bacteria inside the infusion device)	(1) Immediately before the end of lipid emulsion drip	(2) Total number of bacteria inside the infusion device (after completion of TPN infusion + the number of residual viable bacteria inside the infusion device)
None	1	168	129 (18+111)	224	8,989 (1,377+7,612)	330	29,397 (13,929+15,468 )
	2	152	440 (43+397)	25	4,844 (4,175+669)	11	2,980 (958+2,022)
	3	47	48 (9+39)	13	3,313 (268+3,045)	32	16,927 (2,320+14,607)
	4	77	386 (325+61)	618	26,376 (3,780+22,596)	224	22,128 (3,177+18,951)
	5	73	64 (19+45)	668	83,188 (45,504+37,684)	370	43,826 (18,808+25,018)
	**Average±SD**	**103 ± 48**	**213 ± 166 (83+131)**	**310 ± 283**	**25,342 ± 30,067 (11,021+14,321)**	**193 ± 148**	**23,052 ± 13,514 (7,838+15,213)**
5 mL	1	59	321 (112+209)	346	6 (0+6)	181	539 (37+502)
	2	332	34 (6+28)	219	7,038 (1,682+5,356)	159	128 (50+78)
	3	22	684 (144+540)	120	0 (0+0)	33	0 (0+0)
	4	41	89 (22+67)	201	1 (0+1)	20	16 (9+7)
	5	70	0 (0+0)	328	1,120 (29+1,091)	239	19 (6+13)
	**Average±SD**	**105 ±115**	**226 ± 255 (57+169)**	**243 ± 84**	**1,633 ± 2,737 (342+1,291)**	**126 ± 86**	**140 ± 204 (20+120)**
10 mL	1	50	124 (45+79)	194	107 (8+99)	316	3 (0+3)
	2	263	1,620 ( 1,178+442)	193	4,050 (282+3,768)	23	0 (0+0)
	3	93	0 (0+0)	4	0 (0+0)	9	0 (0+0)
	4	84	0 (0+0)	6	1,009 (188+821)	281	1 (1+0)
	5	69	0 (0+0)	646	0 (0+0)	385	54 (19+35)
	**Average±SD**	**112±77**	**349 ± 637 (245+104)**	**209 ± 234**	**1,033 ± 1,555 (96+938)**	**203 ± 156**	**12 ± 21 (4+8)**
20 mL	1	231	0 (0+0)	266	0 (0+0)	196	7 (0+7)
	2	180	498 (199+299)	172	0 (0+0)	195	8 (1+7)
	3	16	19 (3+16)	387	0 (0+0)	22	0 (0+0)
	4	59	18 (4+14)	101	1,997 (691+1,306)	23	0 (0+0)
	5	64	0 (0+0)	535	1,688 (254+1,434)	335	76 (13+63)
	**Average±SD**	**110 ± 81**	**107± 196 (41+66)**	**292 ± 155**	**737± 908 (189+548)**	**154 ± 119**	18±29 (3+15)

Unit : CFU. Devices were used as follows: (A) Sure Plug AD 3-way stopcock SA-TR13 (Terumo Corporation, Tokyo, Japan), (B) Top 3-way stopcock SL(360°)-F-CL (Top Corporation, Tokyo, Japan), and (C) BD connecta™ Ref394501 (Nippon Becton Dickinson Company, Ltd., Tokyo, Japan). (1) Immediately before the end of lipid emulsion indicates the time of 90 mL drip after the start of lipid emulsion. (2) The total number of bacteria inside the the infusion device indicates the sum of the viable bacteria count inside the the infusion device 24 h after the start of TPN infusion and the bacteria count inside the infusion device collected by flushing the inside of the infusion device with PBS.

**Table 2:** Washing effect of different volumes of saline flush

The volume of saline flush	Type of infusion device		
	(A)	(B)	(C)
None	0	0	0
5 mL	1	1	1
10 mL	3	2	2
20 mL	2	3	2

Unit : times. Infusion devices were used as follows: (A) Sure Plug AD 3-way stopcock SA-TR13 (Terumo Corporation, Tokyo, Japan), (B) Top 3-way stopcock SL (360°)-F-CL (Top Corporation, Tokyo, Japan), and (C) BD connecta™ Ref394501 (Nippon Becton Dickinson Company, Ltd., Tokyo, Japan). The total number of bacteria inside the infusion device (the sum of the viable bacteria counts inside the infusion device 24 h after the start of TPN infusion and the bacteria count inside the infusion device collected by flushing the inside of the infusion device with PBS) indicates the number of samples with zero out of the five experiments.
